# Topical Application of Lithium Chloride on the Pulp Induces Dentin Regeneration

**DOI:** 10.1371/journal.pone.0121938

**Published:** 2015-03-26

**Authors:** Kazuya Ishimoto, Satoru Hayano, Takeshi Yanagita, Hiroshi Kurosaka, Noriaki Kawanabe, Shinsuke Itoh, Mitsuaki Ono, Takuo Kuboki, Hiroshi Kamioka, Takashi Yamashiro

**Affiliations:** 1 Department of Orthodontics, Okayama University Graduate School of Medicine, Dentistry and Pharmaceutical sciences, Okayama, Japan; 2 Department of Orthodontics, Okayama University Hospital, Okayama, Japan; 3 Department of Oral Rehabilitation and Regenerative Medicine, Okayama University Graduate School of Medicine, Dentistry and Pharmaceutical sciences, Okayama, Japan; Institute for Frontier Medical Sciences, Kyoto University, JAPAN

## Abstract

We herein describe a novel procedure for dentin regeneration that mimics the biological processes of tooth development in nature. The canonical Wnt signaling pathway is an important regulator of the *Dentin sialophosphoprotein* (*Dspp*) expression. Our approach mimics the biological processes underlying tooth development in nature and focuses on the activation of canonical Wnt signaling to trigger the natural process of dentinogenesis. The coronal portion of the dentin and the underlying pulp was removed from the first molars. We applied lithium chloride (LiCl), an activator of canonical Wnt signaling, on the amputated pulp surface to achieve transdifferentiation toward odontoblasts from the surrounding pulpal cells. MicroCT and microscopic analyses demonstrated that the topical application of LiCl induced dentin repair, including the formation of a complete dentin bridge. LiCl-induced dentin is a tubular dentin in which the pulp cells are not embedded within the matrix, as in primary dentin. In contrast, a dentin bridge was not induced in the control group treated with pulp capping with material carriers alone, although osteodentin without tubular formation was induced at a comparatively deeper position from the pulp exposure site. We also evaluated the influence of LiCl on differentiation toward odontoblasts *in vitro*. In the mDP odontoblast cell line, LiCl activated the mRNA expression of *Dspp*, *Axin2* and *Kallikrein 4* (*Klk4*) and downregulated the *Osteopontin* (*Osp*) expression. These results provide a scientific basis for the biomimetic regeneration of dentin using LiCl as a new capping material to activate dentine regeneration.

## Introduction

The use of a stem/progenitor cell-based approach in regenerative medicine is well documented in various tissues and/or organs. However, an efficient tooth regeneration approach has not been achieved in the clinical setting or in *in vivo* studies. Dentin is the major component of the tooth and covers the dental pulp, which produces the inner structure of the dentin matrix. The dental pulp also contains multipotent precursor cell populations and has a regenerative capacity, and reparative dentin is observed beneath sites of decayed and/or fractured dentin in the pulp chamber [1]. When the tooth crown becomes damaged due to caries and/or fractures, the underlying pulp is partially exposed. Direct pulp capping is a current treatment choice for preserving pulp vitality and activating the self-healing capacity of dentin repair [2]. Pulp capping is the placement of a protective dressing over the exposed pulp to allow the endogenous formation of reparative dentin. In the clinical protocol, calcium hydroxide and mineral trioxide aggregate (MTA) is often used as a capping material, and various clinical and radiographic investigations support its use [3]. However, there remains controversy regarding the clinical efficacy of this material, and practical applications are limited [4,5], as, at least in part, these dressing materials do not have direct effects in inducing dentin regeneration. Indeed, if the tooth is further damaged, with critical pulp exposure, the current protocol cannot be used to heal defective conditions in the pulp via dentin self-regeneration [4].

Dentin is similar to bone in terms of its matrix protein composition and abundance of molecular cascades participating in odontoblast differentiation that are shared with osteoblast differentiation [6]. On the other hand, dentin does not undergo remodeling, and odontoblasts secrete dentin throughout their lifespan [6]. In addition, odontoblasts characteristically extend their cellular processes in well-aligned dentin tubules and exhibit cellular polarity [6]. Dentin sialophosphoprotein (DSPP) is specifically abundant in dentin and much less so in bone tissues [7–9]. In humans, mutations in DSPP result in defective dentin mineralization [10,11]. In mice, *Dspp null* mutant molars display a reduced dentin thickness and hypomineralization [12]. Odontoblasts in *Dspp* mutants also lack the cellular polarity and organization typically observed in wild-type odontoblasts, and aberrant odontoblasts can become trapped in mineralized tissue [12]. Such defective phenotypes are evident only in dentin, not in bone. These findings clearly indicate that Dspp provides dentin-specific features in odontoblasts.

Regarding the molecular regulatory mechanisms associated with the *Dspp* expression, the odontoblast layer demonstrates a high *TOPGAL* and *Axin2* reporter activity, suggesting that the canonical Wnt pathway is involved in both dentinogenesis and in dentin regeneration [13–16]. In addition, we previously found that the *Dspp* expression is activated by WNT10a *in vitro* [17]. We also demonstrated that the double null mutation of Sulf1 and Sulf2 genes, both of which encode endosulfatase and modify the affinity of the cell surface HSPGs for Wnt ligands, exhibited a defective dentin matrix formation with downregulated Wnt canonical signaling pathway [18]. In humans, mutations in *Axin2* and *Wnt10a* cause tooth agenesis and/or hypodontia [19–21]. Recent studies, including ours, have provided direct evidence that Wnt canonical signaling is involved in the promotion of *Dspp* mRNA expression. The canonical Wnt signaling pathway can be activated by LiCl, an inhibitor of glycogen synthase kinase-3 [22]. Mimicking Wnt signaling with LiCl treatment resulted in the upregulation of *Dspp* expression in odontoblast-cell lines, thus indicating the functional importance of Wnt signaling in *dentinogenesis in vitro* [18,23]. However, the *in vivo* effect of LiCl still remains to be elucidated in dentinogenesis.

Dental pulp cells can differentiate into both osteogenic and odontogenic cells [24]. Calcified tissue is formed in response to pulp capping, termed osteodentin [25]. Although osteodentin originates from the pulp, this tissue has the characteristics of bone, rather than dentin, in terms of its overall morphology. Typically, osteodentin lacks a tubular structure, and the forming cells are not trapped within the matrix. Furthermore, osteodentin often contains tunnel defects that fail to seal areas of underlying pulp exposure [5,26]. Previous efforts have emphasized the efficient formation of mineralized tissue, rather than the induction of real dentin.

In this study, we describe a novel pulp capping material that mimics the biological processes of tooth development in nature. Dental pulp itself contains stem/progenitor cell compartments [27], and our approach was to utilize bioactive that mediate *the* organ-specific *transdifferentiation of* pulp cells by activating the canonical Wnt pathway. In the present study, we topically applied LiCl, an activator of canonical Wnt signaling [22], as a novel capping material in order to activate reparative dentin formation.

## Materials and Methods

### Animals

Forty-five male Sprague Dawley rats that were all 5 weeks of age were randomly divided into an experimental group (n = 30) and a sham-treated control group (n = 15). The animal experiments were performed under the research protocol approved by the Animal Research Committee at Okayama University (OKU-2013284). To minimize animal suffering, the number of animals used was based on the minimum required to obtain statistically valid results.

### Dentin and pulp removal, and the capping procedure

**Fig 1:**
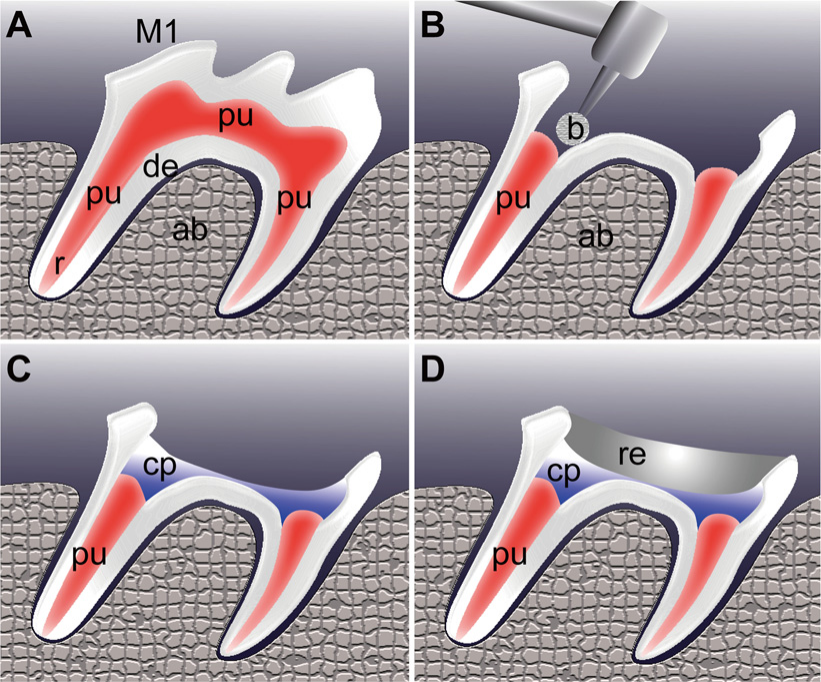
Pulpotomy procedure. The coronal portion of the pulp was removed in the first molars of the 6-week-old Wistar rats. (A) Before pulpotomy. (B) Pulpotomy was performed using a small round bur (b). (C) The wound surface of the sectioned pulps was treated with a LiCl-containing pulp capping agent (cp). A non-LiCl agent was used as a negative control. (D) The cavities were subsequently filled with composite resin (re). Abbreviations: M1, first molar; de, dentin; pu, pulp; ab, alveolar bone; b, bur; cp, capping material; re, resin.

All rats were acclimatized for 1 week before beginning the experiments. At 6 weeks of age, the dentin removal and pulpotomy were performed unilaterally in the maxillary first upper molars (Fig. 1A). The coronal portion of the dentin and the underlying pulp was removed through the occlusal surface using a #1/4 round bur with a low-speed handpiece and sterile saline (Fig. 1B). Bleeding was controlled with light pressure using sterile cotton pellets and paper points before placing the capping materials. In the experimental group of 29 animals, LiCl (Sigma-Aldrich, Poole, UK) was topically applied on the exposed pulp surface (Fig. 1C). LiCl was dissolved in material carriers (macrogol and propylene glycol, 1:1), with a final concentration of LiCl of 10 mM [28]. A mixture of macrogol and propylene glycol is widely applied as a carrier to dissolve mixed antibiotics both in clinical settings and *in vivo* [29,30], because this mixture is viscous, but it has a low surface tension which thus allows it to demonstrate a better diffusion of the medicament [31]. The pulp in the remaining 11 animals was capped with a similar volume of material carrier and employed as a control. Thereafter, the cavity was coated with a bonding agent (*Clearfil Mega Bond*, *Kuraray*, *Japan*) and light cured for 20 seconds. All the cavity was further sealed with light-cured composite resin (UniFil LoFlo, GC, Japan) (Fig. 1D).

The experiments were performed under sodium pentobarbital anesthesia (40 mg/kg, ip), with supplemental doses given to maintain animal anesthesia throughout the experiment. The rats were maintained under a controlled temperature (22±2°C), light dark periods of 12 hours with free access to chow and water ad libitum. After dental treatment, any abnormal animal behaviors such as grooming, locomotion and feeding were not observed.

### Microcomputed tomography (Micro-CT)

The animals were killed four weeks after pulp capping by cervical dislocation. Three-dimensional images of the tooth structure were analyzed using an inspeXio SMX-90CT Microfocus X-ray CT system (Shimadzu, Kyoto, Japan). Briefly, image acquisition was performed at 90 kV and 110 mA. Image reconstruction was carried out using appropriate cross-sections at a spatial resolution of 17 μm, and the resultant images were processed using the three-dimensional reconstruction software program VG Studio MAX 2.0 (Volume Graphics, Heidelberg, Germany).

### Tissue preparation and histology

The tooth and surrounding bone were fixed in 4% paraformaldehyde or tissue fixative at 4°C overnight. The tissues were decalcified in 12.5% EDTA for three weeks, after which they were dehydrated, embedded in paraffin and serially sectioned at 7 μm for hematoxylin and eosin staining. Frozen cross-sections (10 μm) were also prepared for *in situ* hybridization.

### *In situ* hybridization

*In situ* hybridization using digoxigenin-labeled RNA probes was performed as previously described [18]. The preparation of *Dspp* RNA probes has been also described previously [17].

### Cell culture and real-time RT-PCR analysis

The mDP odontoblast-like cell line derived from the embryonic mouse mesenchyme [32] was used. The mDP cells were plated in 6-well plates in Dulbecco's modified Eagle's medium (DMEM) supplemented with 10% fetal bovine serum (FBS) until they reached confluence. The mDP cells were then cultured with or without 10 mm of LiCl for seven days before being processed for the real-time RT-PCR analysis [28].

Total RNA was extracted from the mDP cells using Isogen (Nippon Gene, Tokyo, Japan), according to the manufacturer's protocol. Total RNA was isolated and reverse-transcribed to cDNA using oligo (dT) primer (Takara, Shiga, Japan). For the real-time RT-PCR analysis, cDNA was amplified with Blend-Taq Plus (Toyobo, Osaka, Japan) using a regular thermal cycler. Quantitative real-time PCR was performed in triplicate employing three independent sets of samples. The relative quantity of transcripts was determined using a standard curve and normalized in comparison with the expression of *glyceraldehyde-3-phosphate dehydrogenase gene* (*Gapdh*) mRNA. The sets of synthetic primers used for the amplification were as follows: *Gapdh*, 5´-GTCCCGTAGACAAAATGGTG-3´ (sense) and 5´-CAATGAAGGGGTCGTTGATG-3´ (antisense); mouse *Dspp*, 5´-AGCCGTGGAGATGCTTCTTA-3´ (sense) and 5´-TCACTCTCGCTGTCACCATC-3´ (antisense); mouse *kallikrein 4* (*Klk4*), 5´-TTGCAAACGATCTCATGCTC-3´ (sense) and 5´-TGAGGTGGTACACAGGGTCA-3´ (antisense); mouse Axin2, 5´-CCTTGCCAAAACGGAATG-3´ (sense) and 5´-TTTCGTGGCTGTTGCGTA-3´ (antisense); mouse *alkaline phosphatase (Alp*), 5´-GCTGATCAT TCCCACGTTTT-3´ (sense) and 5´-CTGGGCCTGGTAGTTGTTGT-3´ (antisense); mouse *osteopontin* (*Opn*), 5´-CTGGCTGAATTCTGAGGGACT-3´ (sense) and 5´-CTTCTGAGATGGGTCAGGCA-3´ (antisense).

Each PCR cycle was carried out as previously described [18,24]. Each amplification reaction was performed and checked to ensure the absence of nonspecific PCR products according to a melting curve analysis using the LightCycler^TM^ system (Roche Applied Science, Mannheim, Germany). The relative cDNA copy numbers were computed using data obtained from serial dilutions of representative samples for each target gene.

Comparison of quantitative variables in two groups was performed by the Mann-Whitney *U* test. *P values* less than 0.05 were considered significant. Significance values were calculated using statistical analysis software (JMP version 5, SAS Institute Inc., Japan).

## Results

### Pulp capping experiments

At four weeks after pulpotomy, two mice were died and excluded from the analysis. Ten teeth in the LiCl group and six teeth in the control group were excluded from the study as the resin sealing dropped off, leaving 19 and eight teeth in total for the LiCl and control groups, respectively.

### Micro-CT analysis

**Fig 2:**
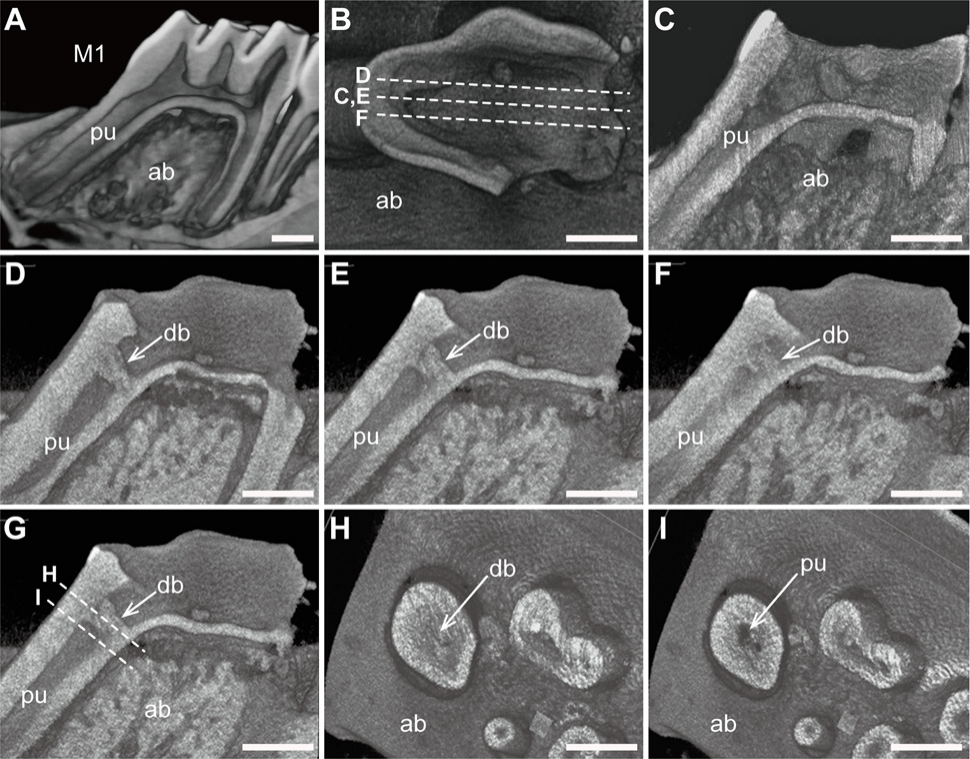
Reconstructed micro-CT images and two-dimensional virtual slices. (A) Three-dimensional reconstructed image of the intact lower first molars in a 10-week-old rat. (B) Virtual sagittal slices were obtained in the control (C) and experimental groups (D-F). (C) No dentin bridges were observed in any of the control mice. (D-F) Series of reconstructed images showing that LiCl treatment induced dentin bridge formation over the exposed pulp surface. (G) Virtual coronal slices were obtained in the experimental group. (H,I) Reconstructed coronal images also illustrated deposition of reparative tissues (H). Abbreviations: M1, first molars; ab, alveolar bone; pu, pulp; db, dentin bridge. Scale bars, 1 mm.

Image reconstruction was conducted to analyze whether dentin bridges were formed over the exposed pulp at four weeks after pulp capping. Following image reconstruction (Fig. 2A), two-dimensional virtual slices were acquired in the sagittal (Fig. 2B-G) and coronal (Fig. 2H,I) planes.

The control group did not show any dentin bridges on the reconstructed sagittal micro-CT images (Fig. 2B). In contrast, 11 of the 19 samples in the LiCl group exhibited complete dentin bridges over the exposed pulp surface (Fig. 2D). Serial reconstructed sagittal images revealed that the dentin bridges were uniform in radiopacity (Fig. 2D-F). Serial sections (Fig. 2G,H) obtained perpendicular to the root axis also demonstrated that the dentin bridges occupied the entire cross-sections of the root chamber (Fig. 2H).

### Histological analysis

**Fig 3:**
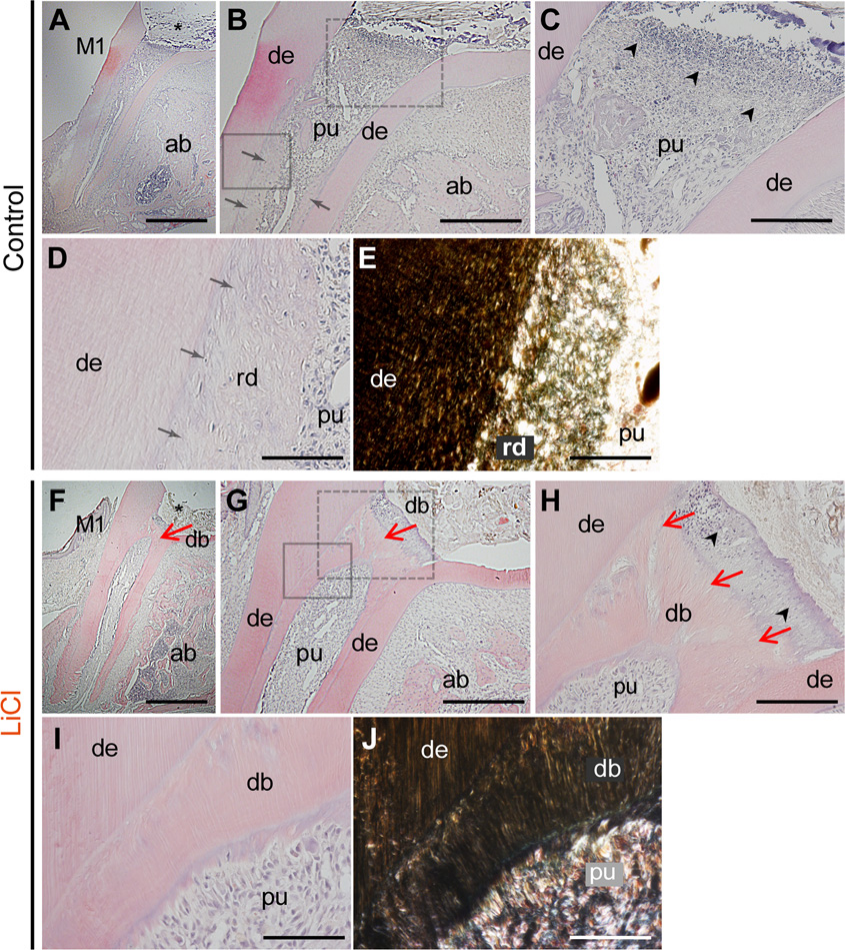
Histological micrographs (H-E) of sagittal sections of the first upper molars. Reparative dentin formation was evaluated on the serial sections in the control (A-E) and LiCl (F-J) groups at four weeks after pulpotomy. (A,F) Pulpotomy was performed in the first molars (M1), and the coronal part of the pulp was removed (asterisk). (B) In the control molars, no reparative dentin bridges were observed beneath the pulp exposure site. However, hard reparative tissue was detected along the residual root dentin surface (arrows). (C) Higher magnification view of the dot box in panel B. Necrotic and disintegrated tissue was observed just beneath the pulp exposure site (arrow heads). (D,E) Bright (D) and dark (E) field images at higher magnification of the solid box in panel B. Dentin tubules were observed in the residual dentin (de), whereas a tubular structure was hardly detected in the deposited reparative tissue (rd). (F-G) The LiCl group exhibited dentin bridges beneath the exposed surface, and the reparative tissue further expanded in the direction of the apex (red arrows). (H) Higher magnification view of the dot box in panel G. A tubular structure was evident in the dentin bridges (red arrows), with only a few cells entrapped in the matrix. (I,J) Bright (I) and dark (J) field images at higher magnification of the solid box in panel G. The reparative matrix in the LiCl group was more condensed, with a well-developed tubular structure, than that observed in the control group. The figure represented the similar results from independent samples. Abbreviations: M1, the first upper molar; mr, mesial root; ab, alveolar bone; de, dentin; pu, pulp; rd, regenerative dentin, db, dentin bridge. Scale bars, 100 μm in A,F; 50 μm in B,G; 20 μm in C,H; 10 μm in D,E,I,J.

Reparative dentin formation was also evaluated histologically in serial sagittal sections obtained four weeks after pulp capping. None of the control group molars capped with material carriers alone showed reparative dentin beneath the region of pulp exposure (Fig. 3A-C). Typically, necrotic and/or disintegrated pulp tissue was observed beneath the area of pulp exposure, with a few particles of the rounded matrix (Fig. 3B,C). Reparative hard tissue was noted along the inner surface of the root with an irregular surface (arrows in Fig. 3B,D). A tubular structure was hardly detected in the reparative tissues, as observed on bright and dark field micrographs (Fig. 3B,D), and some cells were entrapped in the regenerated matrix (Fig. 3D).

In contrast, the LiCl group demonstrated dentin bridges at the exposed surface, and the reparative tissue further expanded along the root surface (red arrows in Fig. 3F,G). At high magnification, a tubular structure was evident in the dentin bridges, with only a few cells entrapped in the matrix (Fig. 3G,H). Furthermore, the reparative matrix in the LiCl group was more condensed, with a well-developed tubular structure, than that seen in the control group, as revealed on bright and dark field micrographs (Fig. 3I,J). These findings indicate that the LiCl-induced reparative hard tissues possessed characteristics of the dentin rather than bone.

### *Dspp* mRNA expression *in vivo*

**Fig 4:**
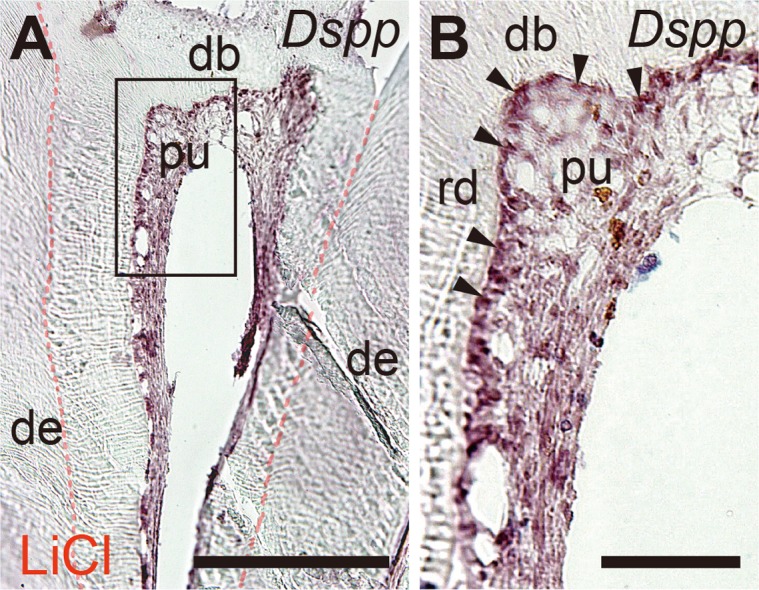
*In situ* hybridization for *Dspp* mRNA in the pulp in the LiCl group. (A) The *Dspp* mRNA expression was confirmed in the pulp cells beneath the dentin bridges. (B) Higher magnification image of the solid box in panel A showing the *Dspp* mRNA expression in the pulp cells. Abbreviations: de, dentin; pu, pulp; rd, regenerative dentin, db, dentin bridge. Arrowheads indicate Dspp transcripts. Scale bars, 50 μm in A; 10 μm in B.

We evaluated the mRNA expression of *Dspp* during regenerative matrix formation *in vivo*. In the LiCl group, the *Dspp* mRNA expression was confirmed in the pulp cells located beneath the dentin bridges (Fig. 4A,B).

### *In vitro* evaluation

**Fig 5:**
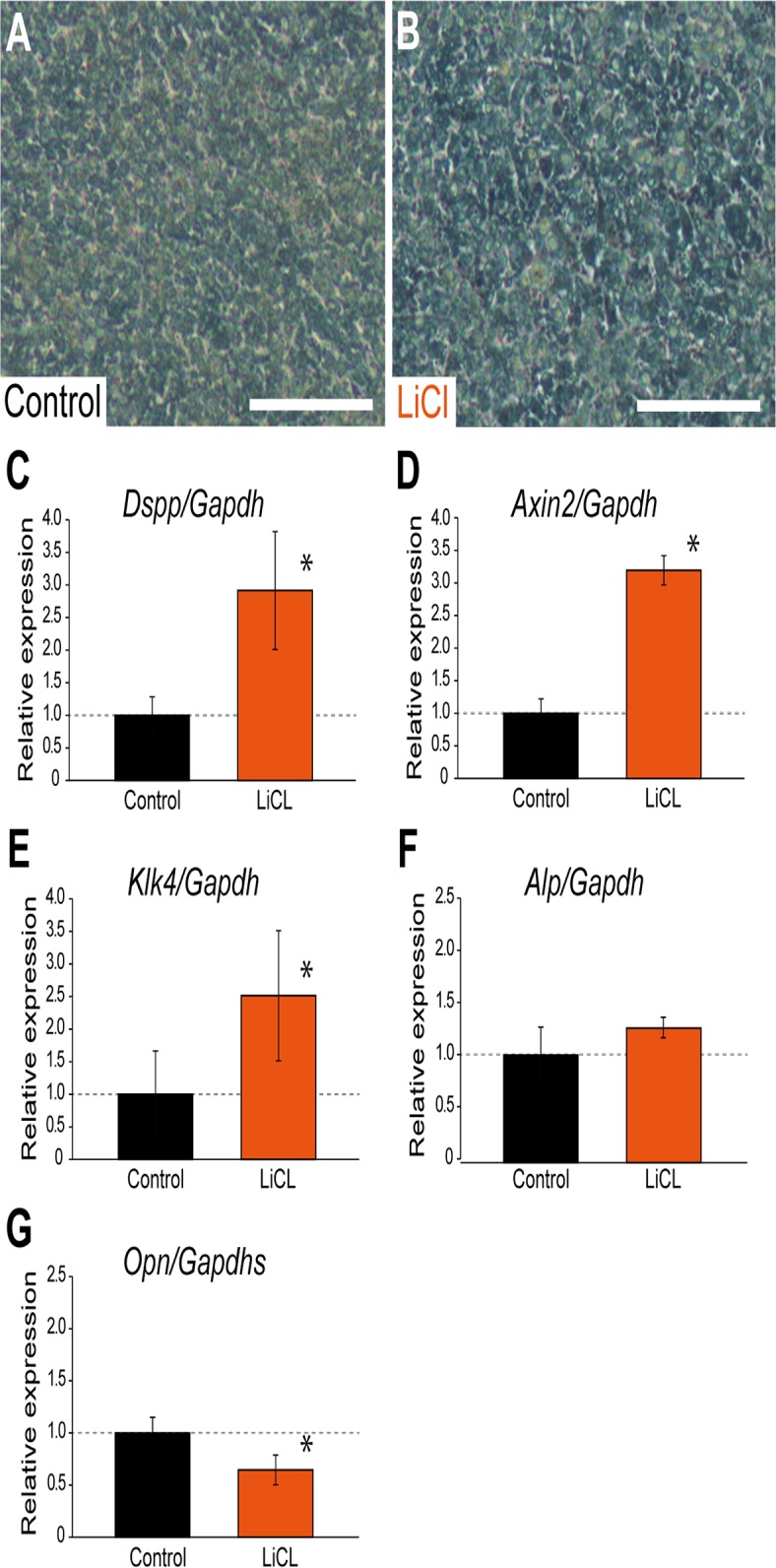
Effects of LiCl on the expression of osteogenic and odontogenic markers. (A and B) mDP were harvested until they reached confluence. The cells were then cultured with (A) or without (B) 10 mM of LiCl for seven days before being processed for the real-time RT-PCR analysis. (C-G) Effects of LiCl on the expression of osteogenic and odontogenic markers on quantitative RT-PCR. Values are the average ± SD of triplicate wells, and are representative of three independent experiments performed. (C-E) LiCl treatment significantly upregulated the expression of the odontogenic markers *Dspp* (*, *p*<0.05; C) and *Klk4* (*, *p*<0.05; E) and the Wnt canonical marker *Axin2* (*, *p*<0.05; D). (F) The *Alp* expression was not affected. (G) In contrast, LiCl treatment significantly downregulated the expression of the osteogenic markers *Opn* (*, *p*<0.05). Scale bars, 100 μm in A and B.

We then attempted to investigate the mechanisms underlying regenerative dentin formation *in vitro*. mDP were harvested until they reached confluence. The cells were then cultured with or without 10 mM of LiCl for seven days before being processed for the real-time RT-PCR analysis. Figure demonstrated microscopic view of the mDP cells before and after LiCl treatment and LiCl treatment did not affect the appearance of mDP cells (Fig. 5A,B).

Real-time PCR demonstrated that LiCl significantly upregulated the *Dspp* and *Axin2* mRNA expression at a dose of 10 mM (*p*<0.05; Fig. 5C,D), as shown in previous study [18]. LiCl application also activated the *Klk4* mRNA expression *significantly* (*p*<0.05; Fig. 5E). Klk4 is a serine proteinase characteristically expressed by odontoblasts and ameloblasts in a stage-specific manner [33,34]. In contrast, LiCl did not change the *Alp* mRNA expression levels (Fig. 5F) and LiCl significantly downregulated the mRNA expression of the osteogenic marker *Opn* (*p*<0.05; Fig. 5G).

## Discussion

This study employed a biomimicry approach using the topical application of LiCl on the amputated pulp surface to achieve transdifferentiation toward odontoblasts from surrounding progenitor cells and subsequent dentin regeneration. This work highlights the potential of this novel pulp capping approach, which mimics and activates the molecular mechanisms of dentine regeneration.

Our approach mimics the biological processes underlying tooth development in nature and focuses on the activation of canonical Wnt signaling to trigger the natural process of dentinogenesis. It has been established that Dspp is a key molecule for dentinogenesis [11,12], and the *Dspp* expression is regulated, at least in part, via the Wnt canonical pathway [17,18] *in vitro*. The importance of Wnt signaling is also supported by genetic studies of human agenesis and/or hypodontia caused by mutations in *Axin2* and *Wnt10a* [19,20]. The present capping procedure using LiCl treatment efficiently induced the formation of dentin bridges just beneath the treated regions. Furthermore, the regenerated tissues exhibited dentin characteristics with well-developed tubular formation. These results provide *in vivo* evidence that LiCl may be used a new tool for efficiently achieving dentin regeneration as a novel treatment approach.

The disadvantages of the previous capping procedure is that the technique resulted in the regeneration of hard tissue, not dentin-like tissue, although osteodentin has a bone-like structure that is more fragile than that of dentin [35]. Such findings are supported by a recent *in vitro* study that clearly showed that traumatic pulp exposure activates the osteogenic potential, while downregulating the odontogenic capacity, *in vitro* [28]. In a previous study, BMPs, such as BMP2, BMP7 and GDF11, were evaluated as possible bioinductive materials activating the self-healing response in the pulp [36]. Such application typically induces the formation of mineralized tissue on the exposed pulp surface; however, the induced matrix is not like that of dentin, but rather that of a bone-like tissue called osteodentin. Since these BMPs activate the *Runx2* expression, it is conceivable that the use of BMPs induces osteogenic commitment during the self-regeneration process and consequent osteodentin formation. Interestingly, the odontoblast-specific overexpression of *Runx2* results in defective phenotypes of dentin formation [37], and the *Runx2* mRNA expression is remarkably downregulated in tooth development, with differentiation to mature odontoblasts [38]. These findings suggest that Runx2 is involved in the cell fate determination of pulpal mesenchymal progenitors regarding whether to commit to the osteogenic lineage or odontogenic lineage and that the downregulation of Runx2 is important for odontogenic commitment.

A recent study demonstrated that the expression of *Axin2*, a marker of the Wnt canonical pathway, has counteracting effects on the *Runx2* expression in the cranial sutures [39]. In the present study, although we found that activation of the Wnt pathway downregulated the osteogenic marker of *Opn* in the odontoblast cell line, the odontoblast line of mDP cells did not express *Runx2* (*Osf2*). Further studies are therefore needed to elucidate the possible associations between the *Runx2* expression and activation of the canonical Wnt signaling pathway in the process of odontoblast differentiation.

The use of a stem/progenitor cell-based approach in regenerative medicine is well documented [40]. The efficacy of autologous transplantation of stem cells has been investigated with respect to dentin regeneration [41]; however, the dental pulp itself contains stem/progenitor cells [27,42], which serve as available donor sources for stem/progenitor cell engraftment into host tissues. Hence, an endogenous cell homing approach, which stimulates self-healing mechanisms using cells already present in the tissues, has the potential to provide novel therapeutic options for dentin regeneration. Regarding efficient dentin regenerations, LiCl has great potential as a bioactive mediating organ-specific transdifferentiation.
